# The effect of injection and topical application of hCG and GnRH agonist to induce sperm-release in the roseate frog, *Geocrinia rosea*

**DOI:** 10.1093/conphys/coaa104

**Published:** 2020-12-04

**Authors:** Aimee J Silla, J Dale Roberts, Phillip G Byrne

**Affiliations:** 1 School of Earth, Atmospheric and Life Sciences, University of Wollongong, Wollongong, Northfields Ave, NSW 2522, Australia; 2 School of Biological Sciences and Centre for Evolutionary Biology, University of Western Australia, Stirling Highway, Nedlands, WA 6009, Australia

**Keywords:** amphibian, animal welfare, assisted reproductive technologies, captive breeding, hormone, percutaneous absorption, sperm, spermiation

## Abstract

Reproductive technologies may assist amphibian conservation breeding programs (CBPs) to achieve propagation targets and genetic management goals. However, a trial-and-error approach to protocol refinement has led to few amphibian CBPs routinely employing reproductive technologies with predictable outcomes. Additionally, while injections can be safely administered to amphibians, perceived animal welfare risks, such as injury and disease transmission, warrant the development of alternative hormone administration protocols. The present study investigated the spermiation response of roseate frogs, *Geocrinia rosea*, administered various doses of human chorionic gonadotropin (hCG) and gonadotropin-releasing hormone agonist (GnRH-a) via subcutaneous injection. This study also quantified the spermiation response of frogs administered both hormones via topical application. Total sperm, sperm concentration and sperm viability were assessed over a 12-h period post hormone administration. Males released sperm in response to the injection of hCG (88–100% response; 5, 10 or 20 IU), but all samples collected from males administered hCG topically (100, 100 + DMSO or 200 IU hCG) were aspermic. In contrast, males consistently released sperm in response to both the injection (100% response; 1, 5 or 10 μg), or topical application (80–100% response; 50, 50 + DMSO or 100 μg) of GnRH-a. Overall, the administration of GnRH-a was more effective at inducing spermiation than hCG. Mean total sperm and sperm concentration were highest in response to the optimal topically applied dose of 100 μg GnRH-a (mean total sperm = 2.44 × 10^3^, sperm concentration = 1.48 × 10^5^ sperm/ml). We provide novel evidence that topical application provides a viable alternative to injection for the administration of GnRH-a to induce spermiation in amphibians.

## Teaser

Reproductive technologies have great potential to overcome captive breeding failure and improve the propagation and genetic management of threatened species. Here we report on the development of novel hormone therapies, including the successful use of topical application of reproductive hormones, to induce sperm release in the Australian roseate frog.

## Introduction

The neuroendocrine control of amphibian reproduction, via the hypothalamic–pituitary–gonadal (HPG) axis, shares basic structural and functional characteristics with fish and other tetrapod vertebrates (Vu and Trudeau, 2016). Reproductive behaviours, gamete maturation and release are primarily triggered by the secretion of the neuropeptide gonadotropin-releasing hormone (GnRH) from the anterior preoptic region of the hypothalamus (Silla and Byrne, 2019). The pulsatile release of GnRH stimulates a surge in the synthesis and release of luteinizing hormone (LH) and follicle-stimulating hormone (FSH) from the pars distalis of the pituitary gland. LH and FSH bind to receptors on the gonads, inducing steroidogenesis and stimulating oogenesis and ovulation in reproductively mature females, and spermatogenesis and spermiation in males (Silla and Byrne, 2019). In nature, this hormonal cascade is dependent on appropriate environmental stimuli, including a complex combination of abiotic and biotic triggers.

Replicating the specific combination of environmental stimuli required for successful reproduction in captivity is not a trivial undertaking (Silla and Byrne, 2019). Amphibians display extreme interspecific variation in reproductive strategies, with the number of unique reproductive modes exhibited unrivalled by any other class of tetrapod vertebrate (Haddad and Prado, 2005, Wells, 2007). As a result of this reproductive diversification, the cues required to elicit reproduction are highly species-specific and, coupled with a lack of knowledge of the natural reproductive ecologies of several species, have led to reproductive failure for many amphibians in captivity (Kouba *et al.*, 2009, Silla and Byrne, 2019). Despite the extreme interspecific variation of reproductive strategies exhibited by amphibians, the fundamental neuroendocrine control of reproduction appears to be broadly conserved (Vu and Trudeau, 2016). Consequently, the administration of exogenous hormones can be used to successfully manipulate the HPG axis and stimulate targeted reproductive events (Silla and Byrne, 2019).

Spermiation has been achieved in a wide range of amphibian species via the administration of purified human chorionic gonadotropin (hCG), or synthetic analogues of GnRH (GnRH-a; also referred to as luteinizing hormone-releasing hormone, LHRH-a) (Silla and Byrne, 2019). The administration of GnRH-a recapitulates the hormone pathways of natural GnRH-1 molecules, operating at the level of the pituitary to stimulate the endogenous synthesis and release of LH and FSH (Clulow *et al.*, 2014). Purified hCG targets receptors directly on the gonads, partially mimicking endogenous LH to stimulate testicular activity (Clulow *et al.*, 2014, Silla and Byrne, 2019). In general, GnRH-a is most effective at eliciting a reliable and predicable spermiation response across amphibian species (Clulow *et al.*, 2014, Silla and Byrne, 2019). Conversely, amphibians exhibit a wide range of hCG sensitivities, as this mammalian gonadotropin is only distantly related to amphibian LH beta units (Clulow *et al.*, 2014). Nevertheless, a growing number of species have been shown to respond more favourably to hCG than GnRH-a (Clulow *et al.*, 2018, Kouba *et al.*, 2012, Silla *et al.*, 2019, Silla and Roberts, 2012). At present, the capacity to predict spermiation responses in novel species is limited, and dose-response curves for both GnRH-a and hCG should be established to optimize spermiation responses (Silla *et al.*, 2019).

Once hormonal induction of spermiation has been achieved, there is enormous potential for additional reproductive technologies to be applied to conservation breeding programs (CBPs) to more effectively reach propagation targets and genetic management goals (Silla and Byrne, 2019). Sperm suspensions can be cryopreserved from captive and wild populations to capture genetic diversity and increase individual reproductive lifespan (Kouba *et al.*, 2013). Either freshly collected or frozen-thawed spermatozoa can then be used to achieve artificial fertilizations (AF), enabling offspring to be generated via crosses between single male–female pairs, or multiple male–female combinations using sophisticated split-clutch factorial mating designs (Byrne *et al.*, 2019, Byrne and Silla, 2020, Silla, 2013, Silla and Byrne, 2019). Reproductive technologies allow ultimate control over breeding designs and have been effectively used to increase the genetic diversity and adaptive potential of captive-bred amphibians (Silla *et al.*, 2018), as well as to test for genetic incompatibilities between individuals from different source populations, which could compromise the success of population augmentation (Byrne and Silla, 2020). Despite such benefits, the adoption of reproductive technologies by amphibian CBPs has been protracted, and these technologies are currently under-utilized on a global scale compared to the use of reproductive technologies for other taxa (Clulow *et al.*, 2014, Silla *et al.*, 2018).

One reason for the slow implementation of amphibian reproductive technologies is that many amphibian CBPs have limited resources and/or lack trained veterinarians with expertise in amphibian injection (Silla *et al.*, 2018). Amphibian injections can be safely and effectively administered, even to very small species, with minimal training and use of sterile consumables (Browne *et al.*, 2019). However, the perception that amphibian injection is associated with animal welfare risks, such as injury and disease transmission, warrants the development of alternative hormone administration techniques. Amphibians possess highly permeable, hypervascularized skin surfaces (Toledo and Jared, 1993), affording the opportunity to investigate the efficacy of epicutaneous administration of reproductive hormones directly to the skin surface (topical application). The present study aimed to empirically test protocols to hormonally induce spermiation in the West Australian roseate frog, *Geocrinia rosea*, as a model for two closely related, threatened species: *G. alba* and *G. vitellina*. Specific objectives were to quantify the effect of (i) varying doses of hCG administered via subcutaneous injection, or topical application, and (ii) varying doses of GnRH-a administered via subcutaneous injection, or topical application, on total sperm, sperm concentration and sperm viability.

## Materials and methods

All procedures were conducted following evaluation and approval by the University of Western Australia’s Animal Ethics Committee (approval numbers RA/3/100/608 and RA/3/100/672). This study was authorized by the Department of Environment and Conservation (DEC), Western Australia (licence number SF 006281).

### Study species

**Figure 1:**
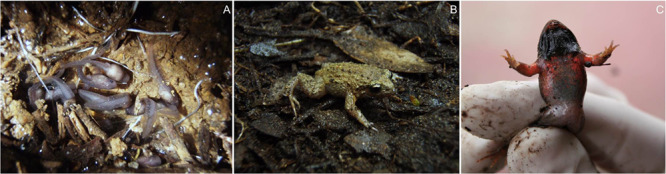
Study species. Images of the roseate frog, *Geocrinia rosea*, showing **A**) larval life-stage (non-feeding tadpoles within the nest), **B**) adult life-stage, dorsolateral view and **C**) adult life-stage, ventral view, of a sexually mature male. Images courtesy of A. J. Silla

*Geocrinia rosea* is a small (19–25 mm, snout-vent length), myobatrachid frog endemic to the Donnelly, Warren, Gadne and Shannon Rice drainages of south-west Western Australia (Wardell-Johnson and Roberts, 1993). Breeding activity typically occurs from September to December (Austral spring to early summer) in temperate hardwood forests. Female *G. rosea* deposit 26–32 large eggs (mean yolk diameter = 2.35 mm) into small depressions under leaf litter and in rotten tree logs. Embryonic and larval development occurs within the terrestrial nest site, with non-feeding tadpoles hatching into the egg jelly where they remain until metamorphosis (Fig. 1A). This derived breeding biology is characteristic of the four species within the *Geocrinia rosea* complex (*G. alba*, *G. lutea*, *G. rosea* and *G. vitellina*) (Anstis, 2013). This reproductive mode is not shared by other species in the genus *Geocrinia* (*G. laevis*, *G. leai* and *G. victoriana*) or related genera such as *Crinia* or *Pseudophryne*.

### Animal collection and housing

Male *G. rosea* (Fig. 1B) were collected from natural populations located in Beedelup National Park, 335 km south of Perth, Western Australia. Collections took place over two consecutive breeding seasons, during November 2006 (Experiment 1: hCG administration) and November 2007 (Experiment 2: GnRH-a administration). A total of 140 males were collected by tracking their vocalizations, detecting the terrestrial nest site and removing the resident by hand. All males captured were observed broadcasting advertisement calls, and they also exhibited pigmented vocal sacs (Fig. 1C), indicating that they were reproductively mature. Frogs were transported to the University of Western Australia, Crawley, where they were housed individually in plastic aquaria (120 mm D × 80 mm H), with soil and leaf litter sourced from their collection site provided as a substrate. Frogs were housed in an artificially illuminated constant temperature room maintained on a 14/9-h light/dark cycle and a temperature of 15°C. Experiments commenced following an acclimation period of 7 days, imposed in order to minimize the potential effects of collection stress on the efficacy of hormone treatment.

### Experiment 1: effect of hCG administration on spermiation responses

To determine the effect of hCG dose and route of administration on spermiation, 64 frogs were assigned to one of eight experimental treatments (*n* = 8 per treatment); 0, 5, 10 or 20 IU hCG administered via subcutaneous injection, or 0, 100, 100 + DMSO or 200 IU hCG via topical application. Note that frogs were exposed to one experimental treatment only and were not reused during the course of the present study, nor were they used for any experiments prior to or after the experiments presented herein. Frogs were randomly assigned to experimental treatments, and no significant difference (Kruskal–Wallis test: *P* > 0.05) in the weight of animals was detected between treatment groups (mean mass ± SEM (g) = 0.98 ± 0.02). A urine sample was collected from each male prior to the administration of hormones (collection methods detailed below), and in all cases the sample was aspermic. All injections were administered as a single dose subcutaneously into the dorsal lymph sac using ultra-fine 30-gauge needles. All injected hormones were diluted in Simplified Amphibian Ringer (113 mM NaCl, 2 mM KCl, 1.35 mM CaCl_2_, 1.2 mM NaHCO_3_; 274 mOsm kg^−1^) to yield the final hCG dose (0, 5, 10 or 20 IU hCG, Chorulon®) in 100 μL of solution. Frogs administered hCG topically received the appropriate dose of hCG (0, 100, 100 + DMSO or 200 IU hCG) drop-wise to the ventral pelvic region (total application time ~60 s). All topically applied hormones were diluted in 100 μL of Millipore water (MQ H_2_O; 3 mOsm kg^−1^), used as an alternative to SAR in order to promote hormone uptake across the epidermis. One dose of topically applied hCG (100 IU, diluted in MQ H_2_O) was administered in combination with the penetration enhancer DMSO (10% vol/vol). Hormone solutions appeared to be rapidly absorbed, and no excess solution spilled over the ventral abdomen surface (as has been observed in other species). Following hormone administration, frogs were returned to individual holding tanks (40 mm D × 70 mm H). One hour post administration (PA), three layers of sponge (40 mm W × 40 mm L × 3 mm H), moistened with distilled water, were placed in each container. The addition of sponge to each holding container was delayed to allow any hormone solution remaining on the skin surface of each frog to continue to be absorbed rather than soaked into the sponge. Hydrating each frog using moistened sponge ensured that urination could be collected from each frog at every sampling period. Mean urine volume collected was 31.45 ± 2.99 μL (range = 4–115 μL). Experiment 1 was conducted from December 4 to 17, 2006, during the species’ natural breeding season.

### Experiment 2: effect of GnRH-a administration on spermiation responses

To determine the effect of GnRH-a dose and route of administration on spermiation, 75 frogs were assigned to one of eight experimental treatments (*n* = 9–10 per treatment): 0, 1, 5 or 10 μg GnRH-a administered via subcutaneous injection, or 0, 50, 50 + DMSO or 100 μg GnRH-a via topical application. Note that frogs were exposed to one experimental treatment only and were not reused during the course of the present study, nor were they used for any experiments prior to or after the experiments presented herein. Frogs were randomly assigned to experimental treatments, and no significant difference (Kruskal–Wallis test: *P* > 0.05) in the weight of animals was detected between treatment groups (mean mass ± SEM (g) = 1.05 ± 0.02). A urine sample was collected from each male prior to the administration of hormones (collection methods detailed below), and in all cases the sample was aspermic. All injections were administered as a single dose subcutaneously into the dorsal lymph sac using ultra-fine 30-gauge needles. All injected hormones were diluted in Simplified Amphibian Ringer (113 mM NaCl, 2 mM KCl, 1.35 mM CaCl_2_, 1.2 mM NaHCO_3_; 274 mOsm kg^−1^) to yield the final GnRH-a dose (0, 1, 5 or 10 μg GnRH-a, Leuprorelin acetate; Lucrin®) in 100 μL of solution. Frogs administered GnRH-a topically received the appropriate dose of GnRH-a (0, 50, 50 + DMSO or 100 μg GnRH-a) drop-wise to the ventral pelvic region (total application time ~60 s). All topically applied hormones were diluted in 100 μL of Millipore water (MQ H_2_O; 3 mOsm kg^−1^), used as an alternative to SAR in order to promote hormone uptake across the epidermis. One dose of topically applied GnRH-a (50 μg, diluted in MQ H_2_O) was administered in combination with the penetration enhancer DMSO (10% vol/vol). Hormone solutions appeared to be rapidly absorbed, and no excess solution spilled over the ventral abdomen surface (as has been observed in other species). Following hormone administration, frogs were returned to individual holding tanks (40 mm diameter × 70 mm deep). One hour post administration (PA), three layers of sponge (40 mm W × 40 mm L × 3 mm H), moistened with distilled water, were placed in each container. The addition of sponge to each holding container was delayed to allow any hormone solution remaining on the skin surface of each frog to continue to be absorbed rather than soaked into the sponge. Hydrating each frog using moistened sponge ensured that urination could be collected from each frog at every sampling period. Mean urine volume collected was 38.73 ± 3.08 μL (mean ± sem). Experiment 2 was conducted on November 15–29, 2007, during the species’ natural breeding season.

### Collection and assessment of spermic urine

Spermic urine samples were obtained at 3, 7 & 12 (± 10 min) hours PA according to standard protocols described previously (Silla, 2010, Silla, 2011, Silla and Roberts, 2012). Samples were collected by inserting the tip of a 50-μL glass microcapillary tube (fire polished and cooled) into the opening of the cloaca until urination was achieved. For each sample, spermic urine volume was measured and the sample immediately prepared for the assessment of sperm yield and sperm viability. Note that sperm viability was the only measure of quality assessed, while sperm motility was observed, motility parameters were not quantified as many samples fell below the minimum volume required for computer assisted sperm analysis (CASA). Spermic urine samples were mixed with 5 μL of a 1:50 dilution of SYBR-14, followed by 2 μL of propidium iodide (Invitrogen, L-7011). The sample was incubated in the dark for 7 min following the addition of each solution. Wet mounts were prepared and the proportion of viable sperm evaluated under a fluorescent microscope at a wavelength of 490 nm. Spermatozoa fluorescing bright green were considered viable (live), while those exhibiting red fluorescence were considered non-viable (dead). The total sperm count and proportion of viable sperm per sample was determined by counting the sperm present in each urine sample in its entirety. Because the exact number of sperm was counted for each sample (note a counting chamber was not used), sperm concentration was calculated as: sperm concentration (sperm/ml) = [total sperm/urine volume (μL)] × 1000. The total sperm released, sperm concentration (sperm/ml) and sperm viability (proportion live/total sperm) for each individual was calculated as the sum of all collection periods (3, 7 & 12 h).

### Testis histology

Transverse sections of the testis were taken to investigate differences in spermatogenic activity across experimental treatments. Immediately following collection of the final spermic urine sample (12 h PA), a random subset of five individuals from the 20-IU hCG injection, 1-μg GnRH-a injection, 100-μg GnRH-a topical treatment and 0-μg GnRH-a (control) treatments were euthanized via double pithing and preserved in 70% ethanol. These hormone treatments were selected as they represent the most effective dose administered via injection and topical application for each hormone type, with a control treatment provided for reference. Preserved specimens were later dissected, and the right testis from each individual was removed, embedded in paraffin wax and serially sectioned. The tissue was stained with Gill’s hematoxylin and eosin and permanently mounted on pathology-grade glass microscope slides (Universal Choice Pty Ltd, Turrella, Australia). Stained testis sections were photographed using a digital camera mounted to a C-mount compound microscope (Leica DFC 295 and Leica 750; Leica Microsystems Pty Ltd, North Ryde, Australia).

**Table 1:** The effect of hCG treatment on spermiation

**Dose**	**Route of administration**	**Males spermiating**	**Total sperm (×10** ^**3**^**)**	**Sperm viability**
**0 IU**	Injection	0/8 ^B^	0.00 ± 0.00 ^C^	-
**5 IU**	Injection	7/8 ^A^	0.19 ± 0.10 ^B^	0.75 ± 0.04 ^A^
**10 IU**	Injection	8/8 ^A^	0.24 ± 0.07 ^B^	0.81 ± 0.05 ^A^
**20 IU**	Injection	8/8 ^A^	0.72 ± 1.23 ^A^	0.84 ± 0.04 ^A^
**0 IU**	Topical	0/8 ^B^	0.00 ± 0.00 ^C^	-
**100 IU**	Topical	0/8 ^B^	0.00 ± 0.00 ^C^	-
**100 IU + DMSO**	Topical	0/8 ^B^	0.00 ± 0.00 ^C^	-
**200 IU**	Topical	0/8 ^B^	0.00 ± 0.00 ^C^	-

Data shown are the number of males spermiating/total number of males, or untransformed mean ± SEM. Data represent total values collected over the three sampling times (3, 7 and 12 h). Data presented were analysed using Fisher’s exact tests (males spermiating), Kruskal–Wallis tests (total sperm), or one-way ANOVA (sperm viability). Letters displayed are the result of post hoc tests. Within a column, treatments that share a letter are not significantly different (*P* > 0.05). See methods for details of all statistical analyses.

### Statistical analyses

The number of males spermiating was compared between hCG treatments (Experiment 1) and GnRH-a treatments (Experiment 2) using two-tailed Fisher’s exact tests. Nonparametric Kruskal–Wallis (KW) tests were used to examine differences in mean total sperm number and sperm concentration. Within each model, the response variable was either total sperm or sperm concentration and treatment was a fixed factor. Post hoc treatment comparisons were made using Wilcoxon matched-pair tests. To assess the effect of sampling time on the number of sperm released, a linear mixed effects (LME) model fitted with restricted maximum likelihood (REML) was performed, where sampling time (3, 7 or 12 h) was a fixed categorical effect, male ID was a random effect and the response variable was the number of sperm released. To assess the effect of hCG treatment and GnRH-a treatment on sperm viability, two separate one-way analyses of variance (ANOVAs) were performed. Sperm viability data (proportions) were arcsine transformed prior to analysis using the transformation sin^−1^(√*x*). To verify homogeneity of variances, Levene’s tests were performed on transformed data. Post hoc treatment comparisons were made using Tukey–Kramer honestly significant difference (HSD) post hoc tests. Regression analyses were used to examine the association between body mass and each of the response variables quantified, because no significant associations were identified, and body mass was not included as a covariate in any of the statistical models. All statistical analyses were performed using JMP 14.1.0 software package (SAS Institute Inc. North Carolina, USE). For all analyses, statistical significance was accepted at *P* < 0.05.

## Results

### Experiment 1: effect of hCG administration on spermiation responses

The percentage of frogs spermiating in response to 5, 10 or 20 IU hCG (88–100%) via injection was significantly greater than the number of frogs spermiating in response to all other dose treatments (0%; Table 1). Hormone treatment had a significant effect on the number of sperm released at each time point (3, 7 and 12 h; *F*_7_ = 15.943, *P* < 0.0001), but there was no effect of time (*F*_2_ = 1.861, *P* = 0.161), nor was there a significant treatment-by-time interaction (*F*_14_ = 1.788, *P* = 0.051). Overall, mean total sperm and sperm concentration differed significantly among treatment groups (total sperm: χ^2^ = 53.62, *P* < 0.0001, sperm concentration: χ^2^ = 53.69, *P* < 0.0001). Mean total sperm released was significantly higher in response to the injection of 20 IU hCG compared with all other treatments (Table 1). Sperm concentration ranged from 0.69 to 3.16 × 10^4^ sperm/ml, and post hoc analysis mirrored the results of mean total sperm. Of the three treatments that elicited a spermiation response (5, 10 and 20 IU hCG), sperm viability of spermic urine samples was statistically similar (*F*_2,22_ = 1.19, *P* = 0.326; Table 1).

**Table 2:** The effect of GnRH-a treatment on spermiation

**Dose**	**Route of administration**	**Males spermiating**	**Total sperm (×10** ^**3**^**)**	**Sperm viability**
**0 μg**	Injection	0/9 ^B^	0.00 ± 0.00 ^C^	-
**1 μg**	Injection	10/10 ^A^	1.42 ± 0.27 ^AB^	0.70 ± 0.03 ^AB^
**5 μg**	Injection	10/10 ^A^	0.82 ± 0.19 ^B^	0.57 ± 0.05 ^B^
**10 μg**	Injection	9/9 ^A^	0.71 ± 0.25 ^B^	0.61 ± 0.06 ^AB^
**0 μg**	Topical	0/9 ^B^	0.00 ± 0.00 ^C^	-
**50 μg**	Topical	9/9 ^A^	1.95 ± 0.57 ^AB^	0.79 ± 0.03 ^A^
**50 μg + DMSO**	Topical	8/10 ^A^	1.28 ± 0.48 ^AB^	0.65 ± 0.06 ^AB^
**100 μg**	Topical	9/9 ^A^	2.44 ± 0.64 ^A^	0.72 ± 0.05 ^AB^

Data shown are the number of males spermiating/total number of males, or untransformed mean ± SEM. Data represent total values collected over the three sampling times (3, 7 and 12 h). Data were analysed using Fisher’s exact tests (males spermiating), Kruskal–Wallis tests (total sperm) or one-way ANOVA (sperm viability). Letters displayed are the result of post hoc tests. Within a column, treatments that share a letter are not significantly different (*P* > 0.05). See methods for details of all statistical analyses.

### Experiment 2: effect of GnRH-a administration on spermiation responses

The percentage of frogs spermiating in response to the administration of 1, 5 or 10 μg GnRH-a via injection (100%), or 50, 50 + DMSO or 100 μg GnRH-a administered topically (80–100%) was significantly greater than frogs administered 0 μg GnRH-a either via injection or topically (0%; Table 2). Between 70 and 100% of males received GnRH-a via injection or topically released sperm and there was no significant difference between the number of males spermiating between treatments (*P* > 0.05). Hormone treatment had a significant effect on the number of sperm released at each time point (3, 7 and 12 h; *F*_7_ = 2.756, *P* = 0.014), but there was no effect of time (F_2_ = 0.406, *P* = 0.667), nor was there a significant treatment-by-time interaction (*F*_14_ = 0.602, *P* = 0.860). Overall, both mean total sperm and sperm concentration differed significantly among treatment groups (total sperm: χ^2^ = 44.90, *P* < 0.0001, sperm concentration: χ^2^ = 43.11, *P* < 0.0001). The topical application of 100 μg GnRH-a elicited the greatest response, with mean total sperm significantly higher than 0, 5 or 10 μg GnRH-a administered via injection (Table 2), but statistically similar to the mean total sperm obtained from frogs administered 50 or 50 + DMSO μg GnRH-a topically, or 1 μg GnRH-a via injection. Sperm concentration ranged from 0.17 to 1.48 × 10^5^ sperm/ml, and post hoc analysis mirrored the results of mean total sperm, with sperm concentration significantly greater in response to the application of 100 μg GnRH-a, compared with 0, 5 or 10 μg GnRH-a administered via injection. Sperm viability also differed significantly among treatments (*F*_5,53_ = 2.96, *P* = 0.021; Table 2), with the topical application of 50 μg GnRH-a generating samples with a higher viability compared to the injection of 5 μg GnRH-a (Table 2).

### Testis histology

**Figure 2:**
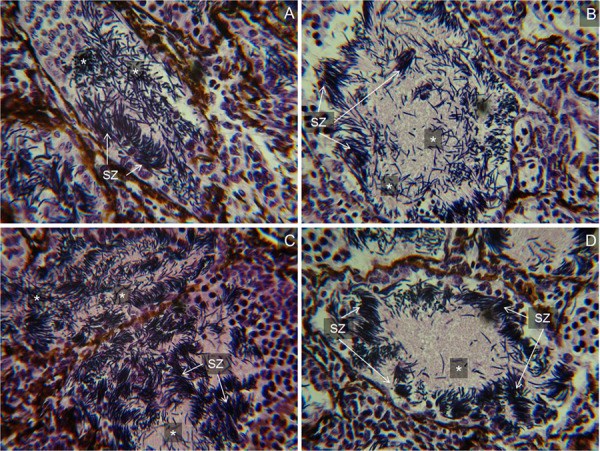
Transverse section of the testis. Representative images of histological sections of testis taken from the following hormone treatments; **A**) 20 IU hCG injection, **B**) 1 μg GnRH-a injection, **C**) 100 μg GnRH-a topical and **D**) 0 μg GnRH-a control. Shown are attached spermatozoa bundles (SZ) and detached spermatozoa (*).

Testis histology revealed high numbers of detached spermatozoa in the testis of frogs receiving hormone treatment (20 IU hCG injection, 1 μg GnRH-a injection and 100 μg GnRH-a topical) compared with very few detached spermatozoa in the testis of control animals (0 μg GnRH-a; Fig. 2). Typically, during spermiation, spermatozoa are detached from sertoli cells and are visible in the lumen and seminiferous tubules of histological sections of the testis. Both hormone-treated and control animals displayed bundles of attached spermatozoa and did not exhibit any signs of sperm depletion (Fig. 2).

## Discussion

To date, amphibian conservation breeding programs have largely adopted a trial-and-error approach to the development of spermiation protocols, with very few studies empirically testing the sperm-release response of amphibians administered hCG or GnRH-a at multiple doses (Kouba *et al.*, 2012, Silla *et al.*, 2019). Here, we quantified the efficacy of varying doses of hCG and GnRH-a (four dose treatments per hormone type) administered using traditional methods of hormone injection. Additionally, we investigated the effect of topical application of both hormones directly to the ventral abdominal surface, as an alternative method of hormone administration to induce gamete-release in amphibians. Collectively, our experiments yielded two main results. First, we demonstrate that spermiation can be successfully induced in *G. rosea* following the administration of both hCG and GnRH-a, but, as expected, these hormones are not equally effective. Second, we demonstrate that topical application of GnRH-a (but not hCG) is effective at eliciting a spermiation response, representing a viable alternative procedure for the administration of this particular hormone.

Results from the present study are consistent with a growing literature indicating species specificity in the spermiation response of amphibians administered hCG and GnRH-a (Clulow *et al.*, 2014, Silla and Byrne, 2019). The optimal total number of sperm released in response to the administration of hCG (via injection) was achieved at a dose of 20 IU, with 100% of males responding to hormone administration, but with relatively low sperm output. The percentage of males responding to the administration of GnRH-a was also maximized, with 100% of males spermiating in response to the injection of 1, 5 or 10 μg, or topical application of 50 or 100 μg GnRH-a. Overall, the average number of sperm released in response to the optimal injected dose of 1 μg GnRH-a, or topically applied dose of 100 μg GnRH-a, was approximately two to three-and-a-half times that of the total released in response to the optimal dose of hCG. Importantly, the total number of sperm released in response to GnRH-a was also less variable compared with hCG (as indicated by a lower mean standard error). These findings are consistent with previous studies indicating that species within the family Myobatrachidae respond more favourably to the administration of GnRH-a (Byrne and Silla, 2010, Silla and Roberts, 2012). It is important to note that numerous forms of synthetic GnRH-a exist with different biological potencies, which should be considered when comparing effective doses between species. However, despite differences in activity profiles between GnRH agonists, overall GnRH-a is regarded as more effective at eliciting a reliable gamete-release response across a range of amphibian species compared to hCG (Kouba *et al.*, 2009, Silla and Byrne, 2019).This is primarily because (i) GnRH-a targets the hypothalamus rather than the testis and therefore triggers a more complete hormonal cascade and (ii) because hCG is only distantly related to amphibian LH beta subunits, leading to more variable responses and higher dose requirements (Clulow *et al.*, 2014, Vu and Trudeau, 2016). Despite this, species belonging to the families Bufonidae, Limnodynastidae and Pelodryadidae respond more favourably to hCG (Clulow *et al.*, 2018, Kouba *et al.*, 2012, Silla *et al.*, 2019, Silla and Roberts, 2012). Research has only recently begun to look for phylogenic patterns in the response of amphibians administered exogenous hormones; however, results indicate that amphibian responses to hormone treatment might be evolutionarily constrained (Silla *et al.*, 2019, Silla and Roberts, 2012). Further comparative research is required in order to confirm existing phylogenic differences in hCG and GnRH-a efficacy, as well as identify additional phylogenetic patterns, which would allow refinement of spermiation protocols to be fast-tracked for novel species.

Sperm viability is an important indicator of fertilization capacity, obtaining sperm samples of maximum viability is therefore an important target for ensuring successful artificial fertilizations. Additionally, it is important that sperm suspensions are of high initial viability prior to cryopreservation, as current freeze-thaw protocols often result in a substantial loss of viability (Poo and Hinkson, 2019). Sperm viability was high in response to the two optimum GnRH-a dose treatments: 1 μg injected or 100 μg GnRH-a topically applied, averaging 70 and 72% live sperm, respectively. However, sperm viability may be further enhanced by reducing sampling intervals and identifying optimal collection times. The present study collected sperm samples at three time points (with up to five hours between collections) for the first 12 h post hormone administration. Sampling intervals were not frequent enough to detect a peak period for sperm collection in our study species. A recent study in the Booroolong frog, *Litoria booroolongensis*, collected samples every 1–2 h over 24 h and clearly identified a narrow peak collection period based on maximum sperm quantity, motility and velocity (Silla *et al.*, 2019), providing an excellent model sampling design for future research. Of note, species-specific differences in peak collection times have been previously identified (Silla and Byrne, 2019, Silla and Roberts, 2012) and are likely to reflect interspecific variation in mating system structure, and associated differences in basal levels of circulating androgens and capacity for sperm production (Silla and Byrne, 2019). Consequently, it is important to identify peak collection periods on a species-specific basis, and target these precise peak periods in order to promote successful biobanking and artificial fertilization.

With a view towards maximizing reproductive output of captive amphibians, it is not uncommon for managers of CBPs to hormonally induce spermiation repeatedly within short intervals (Roth and Obringer, 2003). Testis histology conducted in the present study indicated no signs of sperm depletion following a single induction in *G. rosea*, suggesting that additional hormonal stimulation events could be employed to collect supplementary sperm samples. This approach, however, should be used with caution in order to ensure animal welfare. While empirical research investigating the effects of repeat spermiation induction in amphibians is largely lacking, there is some evidence that frequent hormone administration can negatively impact sperm concentration, sperm motility and male body condition (Arregui *et al.*, 2019, McDonough *et al.*, 2016, Roth and Obringer, 2003). Recent research in the Fowler’s toad (*Anaxyrus* (*Bufo*) *fowleri*) suggests that males should be permitted a minimum recovery period of 2–3 weeks between hormone injections in order to maintain sperm concentration and avoid significant weight loss (McDonough *et al.*, 2016). Importantly, the maximum stimulation frequencies that can be imposed, and recovery periods required to avoid sperm depletion and deleterious effects on animal welfare, are likely to be species-specific and related to the timing and duration of spermatogenesis, which will most likely differ depending on a species’ reproductive mode. Additionally, it is important to note that data are currently not available on the long-term impacts of repeat hormone stimulation on male, or female, reproductive fitness. Repeat hormonal stimulation should therefore be limited, and appropriate recovery periods imposed to limit possible impacts on reproductive lifespan and fecundity of captive amphibians.

An alternative approach to the injection of reproductive hormones is topical application directly to the ventral abdominal surface (epicutaneous administration). The permeability and hypervascularization of amphibian skin promotes the percutaneous absorption of many compounds, including antibiotics, anaesthetics, toxins, hormones and vitamins (Llewelyn *et al.*, 2016). In particular, ventral abdominal skin generally has a thinner epidermis and increased vascularization compared to the dorsum, allowing chemicals to be rapidly absorbed into circulation (Llewelyn *et al.*, 2016). Percutaneous absorption of chemicals across the ventral abdominal skin of amphibians has been successful for chemicals even of high molecular weight (≈ 8 kDA), which are unable to be systemically absorbed through mammalian skin (Llewelyn *et al.*, 2016). However, the extreme molecular weight of hCG (≈ 36 kDA) is the most likely explanation for its lack of effect in the present study, even despite the addition of a penetration enhancer (DMSO). In contrast, the topical application of GnRH-a (MW ≈ 1.2 kDA) was highly successful and offers a viable alternative route of administration to induce spermiation in *G. rosea*, and potentially many other amphibian species. Topical application of GnRH-a has similarly been shown to successfully induce spermiation in male American and Gulf Coast toads (*Anaxyrus americanus* and *Incilius valliceps*; Rowson *et al*., 2001) and has been effective at inducing spawning in male–female pairs of Northern Corroboree frogs (*Pseudophryne pengilleyi*; Silla *et al.*, 2018). Combining GnRH-a with the penetration enhancer DMSO had no effect on the number of males that responded to topical hormone application, the total sperm or sperm concentration released. Additionally, a small number of frogs exhibited skin irritation (redness) at the site of hormone application. Given the lack of beneficial effects and potential welfare concerns reported here and elsewhere (see Rowson *et al*., 2001), further use of DMSO is not advised.

At present, it remains to be validated whether the topical application of reproductive hormones reduces the physiological stress response of amphibians compared to those receiving injections. However, the perceived welfare benefits, including a reduced risk of injury or disease transmission, has led to a number of zoological institutions promoting the development of non-invasive alternatives to amphibian injection, including topical and intranasal deliveries (Julien *et al.*, 2019, Silla *et al.*, 2018). Additionally, the development of non-invasive hormone therapies to induce sperm release is highlighted as one of the primary objectives of the Amphibian Conservation Action Plan (ACAP; a formal document of international significance for the conservation of amphibians) (Della Togna *et al.*, 2020). Importantly, for topical application of reproductive hormones to be effective, doses administered are much higher than those administered via injection, so financial implications need also be considered. Interspecific differences in epidermal thickness, composition and vascularization have been identified (Toledo and Jared, 1993), which may also alter the effectiveness of the topical application of GnRH-a among species. It has been suggested that amphibian species occupying a predominantly terrestrial habitat, which typically have a thinner ventral epidermis and greater vascularization, likely represent the most ideal candidates for topical administration (Llewelyn *et al.*, 2016, Silla *et al.*, 2018). Further research is now required encompassing a greater diversity of amphibian species, in order to ascertain the effectiveness of topical administration of GnRH-a in species of differing reproductive ecologies.

## Conclusions

Incorporating reproductive technologies into conservation breeding programs has the potential to enhance species propagation and genetic management and ultimately contribute to the effective recovery of threatened amphibians worldwide. In recognition of their potential, amphibian reproductive technologies are becoming increasingly adopted. Nevertheless, there remain very few examples of studies empirically testing spermiation responses to the administration of multiple hormones across a range of doses. Therefore, ongoing research is required to optimize protocols. Herein, we report that both GnRH-a and hCG can be effectively used to induce spermiation in *G. rosea*, with the optimal injected dose of 1 μg GnRH-a resulting in the release of a high mean concentration of viable sperm. The present study also quantified the efficacy of topical application of both hCG and GnRH-a to induce gamete release in an amphibian. Results from this study have positive conservation implications, as the topical administration of GnRH-a has been proven effective at inducing the release of viable sperm of comparable numbers to those obtained via hormone injection. Topical application of reproductive hormones provides an alternative to amphibian injection that may promote animal welfare (though this remains to be quantified). Results from our work with *G. rosea* may assist the recovery of two closely related threatened species, *G. alba* and *G. vitellina*, and will inform the conservation breeding programs of other threatened amphibian species globally.

## Funding

This study was funded by RSPCA Australia’s Alan White Scholarship for Animal Welfare, the School of Biological Sciences, UWA, and the Australian Research Council (Linkage Grants LP 140100808 and LP170100351).

## Author contributions

AS and JDR conceived the study and designed the experiments. AS collected and maintained the study animals, performed the experiments and collected all data presented herein. AS and PB performed the statistical analyses. AS wrote the manuscript with input from PB and JDR.
